# Electrolyte imbalance in Asphyxiated term neonates: Incidence, predictors, and outcomes from a prospective cohort study in Northern Uganda

**DOI:** 10.1371/journal.pone.0336549

**Published:** 2026-06-04

**Authors:** Bahari Yusuf, Tom Ediamu, Simon Odoch, Zeinab Issa Ali, Yasin Ahmed H. Abshir, Abdulahi Abdirizak Farah, Abishir Mohamud Hersi, Ahmed Owjama, Hamdi M. Yusuf, Zakaria Abdi Said, Theoneste Hakizimana, Walyeldin Elfakey, Grace Ndeezi

**Affiliations:** 1 Department of Pediatrics and Child Health, Kampala International University, Uganda; 2 Aga Khan Hospital, Pediatrics and Child Health, Mombasa, Kenya; 3 Department of Obstetrics and Gynecology, Kampala International University, Uganda; 4 Department of internal medicine, Kampala International University, Uganda; 5 Faculty of Medicine, University of Bahri, Khartoum, Sudan; 6 Department of Pediatrics and Child Health, Makerere University, Kampala, Uganda; Akershus University Hospital, NORWAY

## Abstract

**Background:**

Birth asphyxia is a major cause of neonatal morbidity and mortality, particularly in low-resource settings. Hypoxia and metabolic derangements that occur during asphyxia predispose neonates to electrolyte abnormalities, which may worsen the clinical course and contribute to poor outcomes. Early recognition and management of such imbalances can improve survival and prevent long-term neurological damage. This study aimed to determine the incidence, predictors, and early outcomes of electrolyte imbalance among term neonates admitted with birth asphyxia in Lira Regional Referral Hospital, Uganda.

**Methods:**

A hospital-based prospective cohort study was conducted among term neonates admitted with birth asphyxia, defined as a 5-minute Apgar score <7. Serum sodium, potassium, and calcium levels were measured at admission, and repeated on days 3, 7, and 14 for those still admitted. Clinical information including maternal and perinatal characteristics was recorded. Modified Poisson regression (using SPSS) was performed to identify independent predictors of electrolyte imbalance. Early outcomes, including mortality and length of hospital stay, were documented within the first 14 days.

**Results:**

A total of 152 neonates were enrolled; 52.6% were male. During follow-up, 42 (29.0%) developed hyponatremia, 29 (19.5%) hyperkalemia, and 31 (21.2%) hypocalcemia. Independent predictors of hyponatremia included low Apgar score (0–3), severe hypoxic ischemic encephalopathy (HIE), prolonged intravenous fluid administration (>48 hours), resuscitation at birth, and dehydration. Convulsions, prolonged intravenous fluids, and resuscitation were significantly associated with hypocalcemia, while hyperkalemia was linked to low birth weight and prolonged intravenous fluids (p < 0.05 for all). Mortality was highest among neonates with hyperkalemia, whereas hypocalcemia was significantly associated with prolonged hospital stay (p < 0.05).

**Conclusion:**

Electrolyte imbalances are common among term neonates with birth asphyxia and hypoxic-ischemic encephalopathy, with hyponatremia, hypocalcemia, and hyperkalemia occurring in nearly one-fifth to one-third of cases. These imbalances are predicted by HIE severity, convulsions, prolonged intravenous fluid therapy, and are associated with increased early neonatal mortality, highlighting the need for routine electrolyte monitoring, standardized intravenous fluid protocols, and timely correction of derangements to improve outcomes.

## Background

Birth asphyxia is one of the most serious neonatal emergencies, contributing substantially to global morbidity and mortality in the early neonatal period [1]. It is defined by the World Health Organization as the inability to initiate and sustain breathing at birth [2], and is clinically identified using a 5-minute Apgar score of less than seven [3]. Globally, birth asphyxia affects between 2–9 neonates per 1,000 live births [4], with an estimated 4–9 million cases reported annually [2]. It is responsible for approximately 900,000 neonatal deaths worldwide each year and accounts for nearly one quarter of neonatal mortality [5]. The burden is disproportionately higher in low- and middle-income countries (LMICs), particularly in Sub-Saharan Africa and South Asia, where more than 98% of neonatal deaths occur [6]. In Uganda, birth asphyxia remains the leading cause of neonatal mortality [7].

The pathophysiological consequences of birth asphyxia are profound and multifactorial. Hypoxia and ischemia impair oxidative metabolism, leading to accumulation of lactic acid, energy failure, and dysfunction of cellular ion pumps [2]. This cascade contributes to hypoxic–ischemic encephalopathy (HIE), renal impairment, myocardial dysfunction, and metabolic derangements [8]. A critical yet often under-recognized complication in this context is electrolyte imbalance, particularly involving sodium, potassium, and calcium [9]. These electrolytes play essential roles in neuronal excitability, cardiac function, neuromuscular stability, and fluid homeostasis [2]. Even subtle derangements can precipitate seizures, arrhythmias, poor perfusion, or prolonged recovery in vulnerable neonates [10].

Hyponatremia in asphyxiated neonates is commonly attributed to inappropriate secretion of antidiuretic hormone and impaired renal tubular reabsorption of sodium [11]. It worsens cerebral edema, increases the risk of convulsions, and is associated with higher stages of HIE [2,10]. Hyperkalemia arises from a shift of potassium from the intracellular to extracellular space due to cell membrane dysfunction, compounded by impaired renal excretion secondary to acute kidney injury [12]. This imbalance significantly increases the risk of fatal cardiac arrhythmias [2]. Hypocalcemia is believed to result from suppression of parathyroid hormone secretion and disrupted placental calcium transfer [13], and is strongly linked to hypotonia, cardiac dysfunction, and seizures [2]. Together, these derangements amplify the vulnerability of asphyxiated neonates, compounding the effects of hypoxia and ischemia.

Evidence from South Asia and the Middle East highlights the high prevalence of these electrolyte imbalances among neonates with asphyxia. Studies in India [10], Bangladesh [11], Pakistan [14], Iraq [5], and Nepal [15] have consistently reported that hyponatremia, hypocalcemia, and hyperkalemia are more common in neonates with severe HIE compared to those with mild or moderate disease. For instance, one study in Nepal reported hyponatremia in 81.8%, hyperkalemia in 90.9%, and hypocalcemia in 45.4% of neonates with severe asphyxia [15]. Similar findings from Bangladesh and India demonstrate that electrolyte abnormalities are not only frequent but also strongly associated with increased mortality and prolonged hospitalization [10,11]. Despite this evidence, data from Sub-Saharan Africa remain sparse, and no study from Uganda had comprehensively examined the incidence, predictors, and outcomes of these derangements among asphyxiated neonates.

In Uganda, routine neonatal care protocols for birth asphyxia largely focus on stabilization, resuscitation, and supportive management. Serum electrolyte monitoring is not consistently integrated into the management of neonates admitted with birth asphyxia, largely due to resource constraints. Observations at Lira Regional Referral Hospital revealed that neonates admitted with low Apgar scores often presented with refractory seizures, severe shock, or prolonged hospital stays, yet electrolyte disturbances were not routinely investigated or corrected. This gap in clinical practice highlights the urgent need for local data to inform guidelines, optimize care, and improve neonatal survival.

This study was therefore conducted to determine the incidence, predictors, and early outcomes of electrolyte imbalance among term neonates admitted with birth asphyxia at Lira Regional Referral Hospital in Northern Uganda. The findings are expected to provide vital evidence to guide routine monitoring, inform neonatal management protocols, and contribute to strategies aimed at reducing neonatal morbidity and mortality in resource-limited settings.

## Methods

### Study design and setting

This was a hospital-based prospective cohort study carried out in the Neonatal Intensive Care Unit (NICU) of Lira Regional Referral Hospital (LRRH) in Northern Uganda. LRRH is a major public referral facility serving districts in the Lango sub-region, including Lira, Amolatar, Apac, Dokolo, Kole, and Oyam. It is located approximately 339 kilometers north of Kampala, Uganda’s capital. The NICU at LRRH receives a large number of neonates admitted with low Apgar scores and birth asphyxia every month, making it an appropriate setting for the present study. Data collection was conducted over a three-month period from 15 March to 15 June 2025**,** following approval from the Kampala International University Research Ethics Committee and administrative clearance from Lira Regional Referral Hospital, during which eligible term neonates were consecutively enrolled and followed up for 14 days.

### Study population

The study population consisted of term neonates admitted to the NICU of LRRH with a clinical diagnosis of birth asphyxia. Birth asphyxia was operationally defined as a 5-minute Apgar score <7. Apgar scores were obtained from delivery room records for inborn neonates and official referral notes for outborn neonates, and were cross-checked at NICU admission prior to enrolment. The absence of routine cord blood gas/cord pH testing in this setting is acknowledged as a limitation.

### Eligibility criteria

Neonates were eligible if they were born at term, admitted to the NICU with a diagnosis of birth asphyxia, and if their parents or guardians provided written informed consent. Neonates with major congenital malformations or those who were born preterm were excluded from the study.

### Sample size determination

Sample size was calculated separately for each objective. For Objective One, the sample size was estimated using Daniel’s formula (n = Z²pq/d²) for a single population proportion. Based on findings from Bangladesh, where 10% of neonates with birth asphyxia had hyperkalemia [16], with p = 0.10, d = 0.05, and Z = 1.96 at 95% confidence, the calculated sample size was 138.

For Objective Two, the open epi online sample size calculator using the Fleiss method was applied, based on results from India showing that electrolyte abnormalities increased with the severity of hypoxic-ischemic encephalopathy (46.6% in stage I and 71.4% in stage III) [17]. At 80% power and 95% confidence, the required sample size was 122.

For Objective Three, the open epi online sample size calculator using the Fleiss method was applied, based on data from Bangladesh showing 60% mortality among neonates with electrolyte derangements compared to 22.6% among those with normal potassium levels [18]. This yielded a sample size of 64, which increased to 71 after adding 10% for potential loss to follow-up.

The largest estimated sample size (n = 138) was adopted to ensure adequate power across all objectives. After adding 10% to account for attrition, the final sample size was 152 neonates. Consecutive enrolment was conducted until the required sample size was attained.

### Study procedure

Upon admission to the NICU, neonates were assessed for eligibility. After obtaining informed consent from the parents or guardians, baseline demographic information and perinatal history were collected using a structured questionnaire. Clinical evaluation was then performed, which included assessment of vital signs, HIE severity was assessed using the validated Thompson score, comparable to the Sarnat system [19]. The Thompson score is designed for bedside applicability and has been widely used in low-resource settings where access to EEG and advanced neuroimaging is limited. All HIE staging assessments were performed by trained clinicians using a standardised Thompson score chart. Prior to study initiation, clinicians received a brief training on the scoring system, and staging was verified by the principal investigator, with discrepant assessments resolved by consensus with the attending paediatrician to ensure interrater reliability. Documentation of complications such as seizures, respiratory distress, shock, or dehydration was ensured. Anthropometric measurements including birth weight and length were taken at the time of enrollment.

Blood samples were obtained for serum electrolyte estimation at admission. Neonates with normal electrolytes at baseline were followed up during hospitalization, and repeat measurements were performed on day 3, day 7, and day 14 for those who remained admitted. Clinical progress was monitored daily, focusing on convulsions, urine output, dehydration status, and intravenous fluid administration. Outcomes were recorded within the first 14 days of admission, including survival, death, and length of hospital stay. A prolonged stay was defined as more than 10 days, corresponding to the 75th percentile of the data. The management of asphyxia followed the Uganda clinical guidelines.

### Laboratory procedure

Approximately 2–3 mL of venous blood was collected under aseptic precautions into plain vacutainer tubes. Samples were allowed to clot and centrifuged to separate serum. Sodium and potassium levels were measured using the ion-selective electrode method, while total calcium levels were measured using the Arsenazo III colorimetric method. Serum albumin was also assayed, and corrected calcium was calculated accordingly to account for hypoalbuminemia. The analysis was carried out in the LRRH central laboratory, which routinely undertakes internal quality control procedures. Normal ranges were defined as sodium 135–145 mEq/L, potassium 3.5–5.5 mEq/L, and calcium 7.6–11.3 mg/dL. Any value outside these ranges was classified as an electrolyte imbalance. To ensure accuracy, equipment was calibrated daily, and 10% of randomly selected samples were re-tested to confirm consistency of results.

### Data management and analysis

Data were initially entered into Microsoft Excel, cleaned, and then exported to SPSS version 25 for statistical analysis. Descriptive statistics were computed to summarize baseline characteristics and the incidence of electrolyte imbalances. Bivariate analysis was conducted to assess associations between potential Predictors and electrolyte disturbances. Variables with a p-value less than 0.2 were included in multivariate analysis using modified Poisson regression with robust error variance to identify independent predictors. For incidence and risk-factor analyses, neonates with abnormal electrolyte values at admission were excluded. Only those with normal baseline values were followed. The outcome was defined as the first documented occurrence of hyponatremia, hyperkalemia, or hypocalcemia within the 14-day follow-up period. Once an abnormality was detected, the neonate was classified as having experienced the event, and subsequent measurements were not used for outcome re-definition. Modified Poisson regression with robust variance was used to analyse associations between baseline factors and incident electrolyte abnormalities. This approach was chosen over logistic regression because the outcomes were binary and relatively common, and direct estimation of adjusted risk ratios was preferred to odds ratios. Only the first incident occurrence per neonate was analysed, so repeated-measures or clustering models were unnecessary. Model dispersion was assessed, and robust variance estimators were used to account for potential overdispersion. Separate models were fitted for hyponatremia, hyperkalemia, and hypocalcemia because these represent distinct physiological outcomes. Candidate predictors were selected based on both clinical relevance and bivariate screening (p < 0.20), with clinically important covariates retained even if marginally non-significant. Multicollinearity was assessed using variance inflation factors (all VIFs < 5), and final models were derived using backward stepwise selection to ensure stability.

The strength of associations was reported using adjusted risk ratios with 95% confidence intervals, and statistical significance was set at a p-value less than 0.05.

### Ethical considerations

Ethical approval for the study was obtained from the Kampala International University Research and Ethics Committee (Ethical approval number: KIU-2024–755.). Administrative clearance was granted by the management of LRRH. Informed consent was obtained from all parents or guardians of eligible neonates prior to enrollment. Confidentiality was maintained by de-identifying participant information, and access to data was restricted to the research team. All neonates identified with electrolyte imbalances received appropriate management in accordance with hospital protocols.

## Results

### Baseline characteristics

A total of 152 neonates with birth asphyxia were enrolled. Slightly over half were male (52.6%). Most had an Apgar score 4–6 (90.8%). Low birth weight (<2,500 g) occurred in 9.2%. Respiratory distress was present in 57.2%. Anuria was uncommon (no urine passed in 3.9%), and shock was documented in 3.9%. The baseline clinical and demographic characteristics of the study participants are summarized in **Table 1**.

**Table 1:** Baseline characteristics of study participants.

Characteristic	Frequency	Percentage
**Age at admission**		
<6 hrs	103	67.8
≥6 hrs	49	32.2
**Sex**		
Male	80	52.6
Female	72	47.4
**DM**		
No	146	96.1
Yes	6	3.9
**Hypertension**		
No	139	91.4
Yes	13	8.6
**Obstetric haemorrhage**		
No	108	71.1
Yes	44	28.9
**Infections in pregnancy**		
No	115	75.7
Yes	37	24.3
**Infection type**		
None	115	75.7
Malaria	24	15.8
UTI	13	8.6
**APGAR score**		
0-3	14	9.2
4-6	138	90.8
**Birth weight**		
<2500	14	9.2
2500-4000	138	90.8
**Resuscitated**		
No	13	8.6
Yes	139	91.4
**Resuscitation duration**		
None	13	8.6
1-5 min	36	23.7
6-10 min	33	21.7
>10 min	70	46.1
**HIE severity**		
Mild	93	61.2
Moderate	33	21.7
Severe	26	17.1
**Respiratory distress**		
No	65	42.8
Yes	87	57.2
**Dehydrated**		
No	133	87.5
Yes	19	12.5
**Neonatal sepsis**		
No	113	74.3
Yes	39	25.7
**Convulsed**		
No	82	53.9
Yes	70	46.1
**Passed urine**		
No	6	3.9
Yes	146	96.1
**Shock**		
No	146	96.1
Yes	6	3.9
**Duration of Iv fluids**		
≤48 hrs	64	42.1
>48 hrs	88	57.9
**Sodium at admission**		
Normal	145	95.4
Low	6	3.9
High	1	0.7
**Potassium at admission**		
Normal	149	98.0
High	2	1.3
Low	1	0.7
**Calcium at admission**		
Normal	146	96.1
Low	5	3.3
High	1	0.7

*DM=Diabetes mellitus, UTI=urinary tract infection, HIE=Hypoxic ischemic encephalopathy*

### Incidence of electrolyte imbalance

At baseline, the majority of neonates had normal electrolyte levels and were followed during hospitalization. For sodium, 145 neonates had normal values at admission, of whom 42 (29.0%) developed hyponatremia during follow-up, with most cases detected on day 3. For potassium, 149 neonates had normal levels initially, and 29 (19.5%) developed hyperkalemia, again predominantly identified on day 3. For calcium, 146 neonates were normal at baseline, and 31 (21.2%) developed hypocalcemia, the majority also first noted on day 3. Overall, these findings demonstrate that electrolyte disturbances are common among asphyxiated neonates, often emerging within the first few days of life, underscoring the need for routine monitoring during this critical period. The incidence of electrolyte imbalance among term neonates with birth asphyxia is presented in **Table 2** below.

**Table 2:** Incidence of electrolyte imbalance among term neonates admitted with birth.

Symptom	Frequency	Percentage
**Sodium (7 abnormal at admission and excluded in the incidence analysis)**		
Hyponatremia, N = 145		
No hyponatremia	103	71.0
Hyponatremia	42	29.0 (95% CI 22.1-36.6)
Day hyponatremia diagnosed		
3^rd^ day	37	25.5
7^th^ day	3	2.1
14^th^ day	2	1.4
**Potassium (3 abnormal at admission and excluded in the incidence analysis))**		
Hyperkalemia, N = 149		
No hyperkalemia	120	80.5
Hyperkalemia	29	19.5 (95% CI 13.4-26.2)
Day hyperkalemia diagnosed		
3^rd^ day	26	17.4
7^th^ day	2	1.3
14^th^ day	1	0.7
**Calcium (6 abnormal at admission and excluded in the incidence analysis)**		
Hypocalcemia, N = 146		
No hypocalcemia	115	78.8
Hypocalcemia	31	21.2 (95% CI 14.4-28.8)
Day hypocalcemia diagnosed		
3^rd^ day	27	18.5
7^th^ day	2	1.4
14^th^ day	2	1.4

*CI = Confidence interval.*

### Predictors for electrolyte imbalance

In the multivariable analysis, several independent predictors of hyponatremia were identified. Neonates with a very low Apgar score (0–3) at 5 minutes were 54.9% more likely to develop hyponatremia compared to those with higher scores. Resuscitation at birth increased the risk by 22.7%, while severe hypoxic-ischemic encephalopathy (HIE) raised the risk by 32.1%. Dehydration was a particularly strong predictor, increasing the likelihood of hyponatremia by 62.7%. Prolonged intravenous fluid administration beyond 48 hours was also significantly associated with hyponatremia, raising the risk by 28.5%.

For hyperkalemia, the independent predictors were low birth weight and prolonged intravenous fluid use. Neonates with a birth weight below 2,500 g had a 35.1% higher risk of developing hyperkalemia, while those who received intravenous fluids for more than 48 hours had a 25.0% increased risk. The majority of neonates who developed hyperkalemia (89.7%) had received intravenous fluids for over 48 hours.

Hypocalcemia was independently associated with resuscitation at birth, convulsions, and prolonged intravenous fluid therapy. Neonates who were resuscitated had a 19.6% higher risk, those with convulsions had a 16.9% increased risk, and those who received intravenous fluids beyond 48 hours had a 28.4% increased risk of developing hypocalcemia. A large proportion of affected neonates had undergone resuscitation (100%) and received prolonged intravenous fluids (77.4%). Independent predictors of hyponatremia, hyperkalemia, and hypocalcemia are summarized in Tables 3–5, respectively.

**Table 3:** Multivariable analysis of predictors of hyponatremia among term neonates admitted with birth asphyxia.

Characteristic	Bivariable analysis			Multivariable analysis		
	cRR	95% CI	P value	aRR	95% CI	P value
APGAR score						
0-3	1.874	1.539-2.282	<0.001	1.549	1.201-1.999	0.001*
4-6	Ref					
Resuscitated						
No	Ref					
Yes	1.263	1.071-1.490	0.005	1.227	1.030-1.463	0.022*
HIE severity						
Mild	Ref					
Moderate	1.140	0.955-1.361	0.146	1.049	0.895-1.229	0.552
Severe	1.771	1.469-2.135	<0.001	1.321	1.078-1.620	0.007*
Dehydrated						
No	Ref					
Yes	1.713	1.382-2.122	<0.001	1.627	1.280-2.066	<0.001*
Duration of IV fluids						
≤48 hrs	Ref					
>48 hrs	1.315	1.149-1.505	<0.001	1.285	1.119-1.495	0.001*

*cRR = Crude rate ratio, aRR = adjusted rate ratio, CI = Confidence interval.*

**Table 4:** Multivariable analysis of Predictors of hyperkalemia among term neonates admitted with birth asphyxia.

Characteristic	Bivariable analysis			Multivariable analysis		
	cRR	95% CI	P value	aRR	95% CI	P value
Obstetric haemorrhage						
No	Ref					
Yes	1.202	1.030-1.404	0.020	1.140	0.994-1.309	0.062
APGAR score						
0-3	1.295	0.991-1.691	0.058	1.297	0.962-1.749	0.087
4-6	Ref					
Birth weight						
<2500	1.401	1.070-1.834	0.014	1.351	1.047-1.743	0.021*
2500-4000	Ref					
Duration of IV fluids						
≤48 hrs	Ref					
>48 hrs	1.279	1.146-1.428	<0.001	1.250	1.079-1.448	0.003*

*cRR = Crude rate ratio, aRR = adjusted rate ratio, CI = Confidence interval.*

**Table 5:** Multivariable analysis of Predictors of hypocalcemia among term neonates admitted with birth asphyxia.

Characteristic	Bivariable analysis			Multivariable analysis		
cRR	95% CI	P value	aRR	95% CI	P value
Resuscitated						
No	Ref					
Yes	1.262	1.175-1.357	<0.001	1.196	1.034-1.383	0.016*
Convulsed						
No	Ref					
Yes	1.280	1.032-1.349	0.016	1.169	1.021-1.337	0.026*
Duration of Iv fluids						
≤48 hrs	Ref					
>48 hrs	1.195	1.055-1.353	0.005	1.284	1.106-1.490	0.006*

*cRR = Crude rate ratio, aRR = adjusted rate ratio, CI = Confidence interval*

### Early outcomes of electrolyte imbalance

Overall mortality was 11.8%. Although mortality was higher across all electrolyte derangements, the difference was only statistically significant for hyperkalemia. Hyponatremia was not significantly associated with mortality. Similarly, hypocalcemia showed no statistically significant association with mortality. below is **Table 6**, which summarizes mortality outcomes.

**Table 6:** Mortality associated with electrolyte imbalance among term neonates admitted with birth asphyxia.

Outcome	Mortality (N = 152)		
Overall	Survived, N = 134	Died, N = 18	P value
**Hyponatremia, N = 145**	**Survived, N = 128**	**Died, N = 17**	0.574
No hyponatremia, N = 103	92(89.3)	11(10.7)	
Hyponatremia, N = 42	36(85.7)	6(14.3)	
**Hyperkalemia, N = 149**	**Survived, N = 133**	**Died, N = 16**	<0.001*
No hyperkalemia, N = 120	116(96.7)	4(3.3)	
Hyperkalemia, N = 29	17(58.6)	12(41.4)	
**Hypocalcemia, N = 146**	**Survived, N = 128**	**Died, N = 18**	0.217
No hypocalcemia, N = 115	103(89.6)	12(10.4)	
Hypocalcemia, N = 31	25(80.6)	6(19.4)	

The proportion of neonates with prolonged hospital stay was higher among those with electrolyte disturbances, but the difference was statistically significant only for hypocalcemia. Neither hyponatremia nor hyperkalemia showed a significant association with prolonged hospital stay. **Table 7** below presents the relationship between electrolyte imbalance and hospital stay duration.

**Table 7:** Hospital stay in relation to electrolyte imbalance among term neonates admitted with birth asphyxia.

Outcome	Prolonged hospital stays among survivors (N = 134)		P value
Overall, N = 134	No, N = 101	Yes, N = 33	
**Hyponatremia, N = 127**	**No, N = 97**	**Yes, N = 30**	0.661
No hyponatremia, N = 85	66(77.6)	19(22.4)	
Hyponatremia, N = 42	31(73.8)	11(26.2)	
**Hyperkalemia, N = 131**	**No, N = 98**	**Yes, N = 33**	0.113
No hyperkalemia, N = 108	84(77.8)	24(22.2)	
Hyperkalemia, N = 23	14(60.9)	9(39.1)	
**Hypocalcemia, N = 128**	**No, N = 95**	**Yes, N = 33**	0.002*
No hypocalcemia, N = 97	79(81.4)	18(18.6)	
Hypocalcemia, N = 31	16(51.6)	15(48.4)	

## Discussion

This prospective cohort study demonstrates that electrolyte imbalances are common among neonates with birth asphyxia in Northern Uganda, with hyponatremia, hypocalcemia, and hyperkalemia occurring in nearly one-fifth to one-third of cases. We found that HIE severity, convulsions, prolonged intravenous fluid therapy, and delayed initiation of feeding were independent predictors of electrolyte imbalance, and that these derangements were associated with increased early neonatal mortality. To our knowledge, this is the first prospective study from Northern Uganda to systematically quantify the incidence, predictors, and outcomes of electrolyte imbalances in asphyxiated neonates using serial measurements and albumin-corrected calcium, highlighting a significant evidence gap in this low-resource setting. Most of these disturbances emerged by day 3 of admission, underscoring the importance of early monitoring.

The incidence observed in our study is consistent with reports from South Asia and the Middle East, where electrolyte abnormalities are common among asphyxiated neonates [5,10,11,14,15]. For example, studies in Bangladesh reported hyponatremia ranging between 28–42% and hypocalcemia in 20–40% of cases, figures that are closely aligned with our findings [18,20,21]. Similar findings were reported in Cameroon where hypocalcaemia occurred in 30.9%, hyponatraemia in 32.4% and hyperkalaemia in 10.0% [22].

However, our incidence of hyperkalemia and hyponatremia was lower than that reported in a Napal study where 81.8% had hyponatremia and 90.9% had hyperkalemia [15]. This discrepancy may reflect differences in disease severity, timing of blood sampling, and local NICU practices such as fluid administration.

In the analysis of predictors, we found that hyponatremia was significantly associated with severe HIE, very low Apgar scores (0–3), dehydration, convulsions, resuscitation at birth, and prolonged intravenous fluid administration. These associations are biologically plausible, as hypoxia impairs renal tubular function and stimulates antidiuretic hormone secretion, leading to water retention and dilutional hyponatremia [15]. Our findings are in agreement with studies from Asia, which demonstrated that hyponatremia correlated with the severity of HIE and was more common in neonates who required resuscitation [15].

Hyperkalemia in our cohort was independently associated with low birth weight and prolonged intravenous fluid therapy. These results are consistent with earlier work from by Kwak et al. who noted that low birth weight neonates were particularly vulnerable to potassium disturbances, partly due to renal immaturity and increased cellular [23].

The link with prolonged intravenous fluids likely reflects impaired excretion and dilutional effects [24]. Importantly, the majority of deaths in our study occurred among neonates with hyperkalemia, reinforcing the clinical significance of this finding. Other studies, have similarly reported that hyperkalemia is the most lethal electrolyte imbalance in birth asphyxia, primarily due to its potential to cause fatal arrhythmias [18].

Hypocalcemia was strongly associated with convulsions, resuscitation, and prolonged intravenous fluid therapy. These associations mirror observations from other studies, where seizures were a common presenting feature of hypocalcemia in neonates [25].

A study conducted by Borkenhagen [25] revealed that Convulsions, or seizures, are a common symptom of neonatal hypocalcemia, which is a condition where there’s low calcium in a newborn’s blood. Low calcium levels make nerve cells abnormally excitable, leading to seizures.

In agreement with our findings, a prospective study was carried out in Bangladesh to assess electrolyte status of newborns who had been suffocated at delivery revealed that throughout the electrolyte derangements, corelated with HIE stage [11]. This is consistent with our study. Also in agreement, a prospective cohort study carried out in India revealed that electrolyte derangements were associated with the degree of birth asphyxia [26]. Similarly, asphyxiated newborns had serum levels of hyponatremia and hypocalcaemia that correlated to the degree of asphyxia [2]. Still comparable, a substantial correlation between the Apgar score and both hyperkalemia and hyponatremia were reported by Injeti et al [27].

With regard to early outcomes, our study demonstrated that overall mortality was 11.8%, with the highest mortality occurring among neonates with hyperkalemia (41.4%). This finding is in agreement with Khondaker et al. in Bangladesh, who reported mortality of 60% among neonates with hyperkalemia compared to 22.6% among those with normal potassium levels [18]. The lethality of hyperkalemia lies in its direct effect on cardiac conduction, predisposing to arrhythmias and sudden cardiac death.

Hypocalcemia, on the other hand, did not significantly increase mortality but was associated with prolonged hospital stay in 48.4% of affected neonates compared to 18.6% among those with normal calcium. Hypocalcemia, neonatal hypocalcemia can cause seizures, tetany, and other complications that require close monitoring and treatment, leading to extended hospital stays. Neonates with hypocalcemia require frequent monitoring of calcium levels, which can prolong hospital stays until they stabilize.

The clinical implications of our findings are important. Electrolyte derangements were frequent, often appeared early, and had distinct impacts on outcomes. Hyperkalemia was the strongest predictor of mortality, while hypocalcemia prolonged hospitalization. These results highlight the need for routine electrolyte monitoring as part of the standard management of neonates with birth asphyxia. Early detection and timely correction could reduce complications, shorten hospital stays, and prevent avoidable deaths. In settings such as Uganda, where routine electrolyte testing is not widely practiced due to resource limitations, our findings provide strong evidence to advocate for inclusion of electrolyte monitoring in neonatal care protocols.

### Limitations

This was a single-center study conducted in the NICU of a regional referral hospital with a high case load of complicated deliveries and referrals; therefore, the findings may not be fully generalisable to the wider population of term neonates with birth asphyxia in Uganda.

The absence of routine arterial blood gas and cord pH testing in our setting necessitated reliance on the 5-minute Apgar score, which, while pragmatic and context-appropriate, may have limited diagnostic precision. Electrolytes were measured at fixed time points (admission, days 3, 7, and 14), and continuous daily monitoring was not feasible. Immediate clinical correction of abnormalities also limited the use of longitudinal methods such as GEE or mixed-effects models. Future studies with denser sampling and larger cohorts should apply longitudinal or time-to-event approaches with time-varying covariates.

### Conclusion

This study demonstrated that electrolyte imbalances are common among term neonates admitted with birth asphyxia at Lira Regional Referral Hospital. Hyponatremia, hyperkalemia, and hypocalcemia were observed in nearly one-fifth to one-third of neonates, with most cases detected on day 3 of admission. Several clinical Predictors were independently associated with these disturbances, including low Apgar scores, severe hypoxic ischemic encephalopathy, convulsions, dehydration, resuscitation at birth, low birth weight, and prolonged intravenous fluid administration. Hyperkalemia was the strongest predictor of mortality, while hypocalcemia significantly prolonged hospital stay. These findings provide compelling local evidence to advocate for the integration of routine electrolyte monitoring particularly within the first 72 hours into the standard management protocol for asphyxiated neonates in this and similar resource-limited settings. Early detection and timely correction of electrolyte derangements represent a critical, feasible step toward reducing preventable neonatal mortality and morbidity in this vulnerable population.

### Recommendations

All neonates admitted with birth asphyxia should be routinely screened for electrolyte imbalances, particularly sodium, potassium, and calcium, during the first days of hospitalization. In resource-limited settings where universal testing may not be feasible, priority should be given to neonates with severe hypoxic ischemic encephalopathy, low Apgar scores, those who required resuscitation, those with convulsions, low birth weight, dehydration, or those receiving intravenous fluids for more than 48 hours. Neonates found with electrolyte imbalances, especially hyperkalemia, should be closely monitored and promptly managed to reduce the risk of death. In addition, clinical protocols and guidelines on intravenous fluid use should be strengthened to minimize iatrogenic electrolyte disturbances. Strengthening routine monitoring of labour with the use of partographs is also essential to reduce the severity of asphyxia and its associated complications. Future research should explore long-term outcomes of electrolyte imbalance in birth asphyxia, including neurodevelopmental delay and cerebral palsy, which were beyond the scope of this study.

### Ethics approval and consent to participate

Ethical approval was obtained from the Kampala International University Research Ethics Committee (Ethical approval number: KIU-2024–755). Written informed consent was obtained from parents/guardians before enrollment.

## Abbreviations

APGAR: Appearance, Pulse, Grimace, Activity, Respiration
BA: Birth Asphyxia
HIE: Hypoxic Ischemic Encephalopathy
IV: Intravenous
NICU: Neonatal Intensive Care Unit
LRRH: Lira Regional Referral Hospital

## References

[1] WHO. World Health Organisation recommendations: intrapartum care for a positive childbirth experience. 2018. http://apps.who.int/bookorders 30070803

[2] Bahatkar K, Aundhakar CD. Electrolyte status and plasma glucose levels in birth asphyxia. J Med Sci. 2021;41(1):17–21. doi: 10.4103/jmedsci.jmedsci_93_20

[3] Moshiro R, Mdoe P, Perlman JM. A global view of neonatal asphyxia and resuscitation. Frontiers in Pediatrics. 2019;7(November):1–6. doi: 10.3389/fped.2019.00489 31850287 PMC6902004

[4] Masood N, Muntiha S, Sharif M, Asghar R. Correlation of serum electrolyte changes with severity of birth asphyxia in newborns. Rawal Med Coll. 2016;20:27–9.

[5] Hasan B, Al-Ani M. Electrolyte disturbance in asphyxiated neonates in maternity hospital in Erbil, Iraq. Med J Babylon. 2019;16(4):331. doi: 10.4103/mjbl.mjbl_52_19

[6] Hug L, Alexander M, You D, Alkema L. National, regional, and global levels and trends in neonatal mortality between 1990 and 2017, with scenario-based projections to 2030: a systematic analysis. Lancet Glob Heal. 2019;7(6):e710–20. doi: 10.1016/S2214-109X(19)30163-9 PMC6527519 31097275

[7] Apio G. Birth asphyxia outcomes and associated factors among newborns admitted to a tertiary hospital in Eastern Uganda: a prospective cohort study. BMC Preg Childbirth. 2025;25(1). doi: 10.1186/s12884-025-07603-2 PMC12023549 40275193

[8] Segar JL, Chock VYL, Harer MW, Selewski DT, Askenazi DJ. Fluid management, electrolytes imbalance and renal management in neonates with neonatal encephalopathy treated with hypothermia. Semin Fetal Neonatal Med. 2021;26(4). doi: 10.1016/j.siny.2021.101261 34140246

[9] Prodhan MS, Azad AZM, Shormin S, Kader MF, Islam MDM, Das MK, et al. Electrolyte abnormalities in patients with perinatal asphyxia. Int J Contemp Pediatr. 2022;10(1):1. doi: 10.18203/2349-3291.ijcp20223331

[10] Ahmed N, Sushma U, Thobbi AN. A case control study to evaluate the electrolyte abnormalities in asphyxiated neonates. Int J Contemp Pediatr. 2023;10(6):898–901. doi: 10.18203/2349-3291.ijcp20231497

[11] Nasrin L, Islam MB, Mir K, Hossain MD, Islam MS, Ahmed MU. An observational study: SARS-CoV-2 vaccination response in chronic kidney disease patients on dialysis. Sch J Appl Med Sci. 2024;12(06):746–54. doi: 10.36347/sjams.2024.v12i06.008

[12] Wosenu L, Worku A, Teshome D, Gelagay A. Determinants of birth asphyxia among live birth newborns in University of Gondar referral hospital, northwest Ethiopia: a case-control study. PLoS One. 2018;7:0203763. doi: 10.1371/journal.pone.0203763 PMC6128623 30192884

[13] R M, Sudeshna MD. Study of electrolyte levels in the cord blood of birth asphyxiated neonates and its correlation with the severity of asphyxia. World J Adv Healthc Res. 2021;5(6):183–6.

[14] Shamaoon M, Razzaq N, Ahsan M, Ahmad A, Maqbool T, Chaudhary AJ. Electrolyte imbalance in neonates with hypoxic ischemic encephalopathy: a single center study. Prof Med J. 2020;27(10):2159–64. doi: 10.29309/tpmj/2020.27.10.4128

[15] Thakur J, Bhatta NK, Singh RR, Poudel P, Lamsal M, Shakya A. Prevalence of electrolyte disturbances in perinatal asphyxia: a prospective study. Ital J Pediatr. 2018;44(1):1–6. doi: 10.1186/s13052-018-0496-7 29784025 PMC5963047

[16] D SK, M T. Electrolyte abnormalities in asphyxiated newborns. Int J Contemp Pediatr. 2018;5(3):1036. doi: 10.18203/2349-3291.ijcp20181537

[17] Vamne A, Bhimte B. Metabolic derangement in birth asphyxia due to cellular injury with reference to mineral metabolism in different stages of hypoxic-ischemic encephalopathy in Central India. Indian J Med Biochem. 2017;21(2):86–90. doi: 10.5005/jp-journals-10054-0027

[18] Khondaker AB, Mohammad AAM, Shyla R, Khondaker MA. Electrolyte imbalance and immediate outcome in asphyxiated neonates: a study in a tertiary care hospital, Dhaka. Sch J Appl Med Sci. 2019;6691:3584–7. doi: 10.36347/sjams.2019.v07i11.019

[19] Gohatre H. Comparison of modified sarnat staging and thompson score in neonatal hypoxic-ischemic encephalopathy. Cureus. 2026. doi: 10.7759/cureus.104350 PMC13033165 41913819

[20] Rahman F, Siddique MAB, Hassan MW, Bari MN, Ahmed F. A study on electrolyte imbalance in asphyxiated neonates. KYAMC J. 2017;7(2):775–9. doi: 10.3329/kyamcj.v7i2.33837

[21] Sumona K, Begum Sharifun N, Shah Alam A, Shabnam S, Yesmin Tanjin J, Prodip M, et al. Impairment of renal function in perinatal asphyxia with hypoxic ischemic encephalopathy in term neonates. Khwaja Yunus Ali Medical College J. 2024;14(04):198–205. doi: 10.3329/kyamcj.v14i04.69526

[22] Tague K. Metabolic and electrolyte disorders during perinatal asphyxia in Yaoundé. Heal Res Afr. 2025;3(August):26–30.

[23] Kwak JR, Gwon M, Lee JH, Park MS, Kim SH. Non-oliguric hyperkalemia in extremely low birth weight infants. Yonsei Med J. 2013;54(3):696–701. doi: 10.3349/ymj.2013.54.3.696 23549817 PMC3635635

[24] Weaver LJ, Travers CP, Ambalavanan N, Askenazi D. Neonatal fluid overload-ignorance is no longer bliss. Pediatr Nephrol. 2023;38(1):47–60. doi: 10.1007/s00467-022-05514-4 35348902 PMC10578312

[25] Borkenhagen JF, Connor EL, Stafstrom CE. Neonatal hypocalcemic seizures due to excessive maternal calcium ingestion. Pediatr Neurol. 2013;48(6):469–71. doi: 10.1016/j.pediatrneurol.2013.02.010 23668874

[26] Acharya A, et al. Clinico-biochemical correlation in birth asphyxia and its effects on outcome. Cureus. 2020;12(11). doi: 10.7759/cureus.11407 PMC7725445 33312805

[27] Injeti G, Kher A, Taksande A, Panwar AS. Determination of serum electrolyte and calcium abnormalities in neonates with birth asphyxia. J Pharm Res Int. 2021;:807–15. doi: 10.9734/jpri/2021/v33i60B34683

